# Hospital Reform

**Published:** 1897-11-20

**Authors:** 


The Hospital

A Journal of

The Medical Sciences and Hospital Administration.

Vol. XXIII.—No. 582.] Edited by Sir Henry Burdett, K.C.B., and Solomon C. Smith, M.D., M.R.C.P. [Nov. 20, 1897.

### Hospital Reform.

Our esteemed contemporary, the *Lancet*, has thought fit to make an onslaught upon Sir Henry Burdett with reference to his observations at a recent meeting held for the promotion of hospital reform, and has set forth, in solemn language, that it "will become a serious question whether medical men should be asked to appear on the same platform with him again" in relation to this subject. The "again" seems somehow to have lost its way in the sentence; but that is a minor consideration, more especially in the columns of the *Lancet*. Sir Henry's offence, as far as we can ascertain from the full report given in our issue to-day, seems to have depended upon his statement that many out-patients were found by hospital officers to be in a condition suggestive of inadequate treatment from some general practitioner; whereupon Dr. Ward Cousins "traversed" the very different statement "that patients preferred to go to the hospitals because of the want of skill of the general practitioner," and the *Lancet*, not to be outdone in playing to the gallery, has endeavoured to "go one better" in its distortion of the argument, and has replied to Sir Henry's consequent and very natural expostulation by a magnificent announcement of its determination to "stand by the sentiments" expressed in its leading article. Sir Henry, according to our latest information, is as well as can be expected; and will probably be quite equal to the task of dealing with the *Lancet* according to its deserts. Our own interest in the question passes wholly beyond its merely personal aspects, and leads us to consider its bearings by the light of common sense and experience.

The contention of the *Lancet* and of Dr. Ward Cousins would appear to be, as far as we can understand it, that the general practitioners to whom the poor, as represented by out-patients, habitually resort, are just as learned, just as skilful, and just as painstaking—the last being, perhaps, the most important qualification of the three—as the assistant physicians and assistant surgeons of great hospitals; insomuch that the aforesaid out-patients have nothing to gain with regard to the diagnosis or the treatment of their diseases, and have only the saving of fees to set against the increased expenditure of time which resort to a hospital entails upon them. Of course, if this be so, it leaves no excuse for the continued existence of out patient departments; and it indicates that the only benefit to be obtained from a hospital depends upon the shelter of its wards and the skill of its nurses.

There is an old story of a Quaker who, in the prim speech of his sect, used the word "likewise,' and was rebuked by some Dr. Ward Cousins among his audience, who said: "I suppose you mean 'also?'" "Nay, friend," said the Quaker, "I mean 'likewise.'" "But they are the same," objected the doctor. "Nay, friend, they are not. Sir Benjamin Brodie is a surgeon. Thou art a surgeon 'also,' but not 'likewise.'" The organisation of the great hospitals has been steadily directed, now during many years, towards securing for their staffs the best men educated in the respective schools, the men whose capacities and leanings are best calculated to render them skilful and successful practitioners, while the men of less marked capacity have been suffered to drift away. Of these, again, it seems probable that the more capable will, as a rule, make their mark in private practice of the more remunerative description, and that the less capable will ordinarily be found in possession of the dispensaries and open surgeries, to which persons of the out-patient class largely resort for bottles of physic, which they obtain in return for some very moderate payment. Is it reasonable, is it in harmony with the teachings of experience in any department of human activity, that the work done by the last-mentioned class should be in all respects as good as that done by the first? We think not, although, of course, there will be exceptions. Mr. George Brown, for example, told the meeting of his own successes in rectifying the errors of the staff of St. Bartholomew's; but, then, such practitioners as Mr. George Brown are not to be found in every inferior street in the metropolis. As a rule, there can be no question in the mind of any reasonable person, that the examination made at a great hospital is likely to be more skilful and more complete, even if it were only on account of the presence of students, or that the treatment is likely to be better directed, than that of the private dispensary at which, between certain specified hours, patients may obtain advice and medicine for sixpence.

If this be the case, one of two consequences must necessarily follow. Either the statement made by Sir Henry Burdett must be true, and patients must sometimes present themselves in conditions which are at least partly due to inadequate treatment, or else inadequate treatment does no harm, even negatively, and sick folk are not really the worse for having been the subjects of erroneous opinions and of misdirected medication. The *Lancet* will admit, we suppose, that there must be a difference of some kind between a good doctor and an inferior one, but maintains, as a noble sentiment by which it will "stand," that this difference, whatever it may be, is not of a nature to be prejudicial to the patient.

